# Strengthening the role and functions of nursing staff in inpatient stroke rehabilitation: developing a complex intervention using the Behaviour Change Wheel

**DOI:** 10.1080/17482631.2017.1392218

**Published:** 2017-10-31

**Authors:** Mia Ingerslev Loft, Bente Martinsen, Bente Appel Esbensen, Lone L. Mathiesen, Helle K. Iversen, Ingrid Poulsen

**Affiliations:** a Department of Neurology, Rigshospitalet, Copenhagen University Hospital, Glostrup, Denmark; b Department of Public Health, Section of Nursing, Aarhus University, Aarhus, Denmark; c Copenhagen Centre for Arthritis Research (COPECARE), Centre for Rheumatology and Spine Diseases, VRR Head and Orthopaedics Centre, Glostrup, Rigshospitalet, Denmark; d Department of Clinical Medicine, Faculty of Health and Medical Sciences, University of Copenhagen, Copenhagen, Denmark; e Research Unit on Brain Injury Rehabilitation Copenhagen (RuBRIC), Clinic of Neurorehabilitaion, Hvidovre, TBI unit Rigshospitalet, Denmark

**Keywords:** Behaviour change, complex intervention, nursing, rehabilitation, stroke

## Abstract

**Purpose:** Over the past two decades, attempts have been made to describe the nurse’s role and functions in the inpatient stroke rehabilitation; however, the nursing contribution is neither clear nor well-defined. Previous studies have highlighted the need for research aimed at developing interventions in the neuro-nursing area. The objective of this paper was to describe the development of a nursing intervention aimed at optimising the inpatient rehabilitation of stroke patients by strengthening the role and functions of nursing staff.

**Method:** A systematic approach was used, consistent with the framework for developing and evaluating complex interventions by the UK’s Medical Research Council (MRC). Based on qualitative methods and using the Behaviour Change Wheel’s (BCW) stepwise approach, we sought behaviours related to nursing staffs’ roles and functions.

**Results:** We conducted a behavioural analysis to explain why nursing staff were or were not engaged in these behaviours. The nursing staff’s Capability, Opportunity and Motivation were analysed with regard to working systematically with a rehabilitative approach and working deliberately and systematically with the patient’s goals.

**Conclusion:** We developed the educational intervention Rehabilitation 24/7. Following the MRC and the BCW frameworks is resource-consuming, but offers a way of developing a practical, well-structured intervention that is theory- and evidence based.

## Background

Stroke is the second leading cause of disability worldwide (Feigin, Roth, Naghavi, & Parmer, 2016). Patients who had suffered a stroke experience challenges in relation to everyday tasks due to complications related to, e.g., motor impairment and cognitive deficit. Hence difficulties in relation to walking, eating, communicating, and structuring daily life can be the consequences (Langhorne, Bernhardt, & Kwakkel, 2011). It is evident that patients admitted to organized stroke units are more likely to be alive, independent, and living at home 1 y after the stroke (Organised inpatient (stroke unit) care for stroke, 2013; Winstein et al., 2016). There is evidence for a multidisciplinary effort in stroke inpatient rehabilitation (National Health Board, 2011). In this multidisciplinary team the nurse has traditionally been described as a natural and significant member of the rehabilitation team (Clarke, 2014). However, the manner and mode of a nurse’s role has been difficult to describe. The role and function of nurses in stroke rehabilitation have come under increased focus during the past two decades, with several suggestions and discussions occurring to attempt to clarify them (Long, Kneafsey et al. 2002, Kirkevold 1997, Aadal, Angel, Dreyer, Langhorn, & Pedersen, 2013). The nursing contribution has been described as vague and unclear by nurses, interdisciplinary collaborators, patients, and relatives. Some sources state that nurses believe that rehabilitation is something distinct from care. The lack of a clearly defined role may cause challenges for interdisciplinary collaboration, as a foundation for a strong and fruitful collaboration is a strong understanding of each member’s professional contribution and identity (Johnson, 2015). Furthermore, the lack of a clear contribution can be challenging when collaborating with patients and relatives.

To clarify the contribution of the nurse, a European competence profile described the role of the nurse in rehabilitation as providing teaching, dissemination of information and supervision in the areas of physical, instrumental, and psychological care (Keeken van, Woert van der et al. 2007). Kirkevold, based on empirical studies, developed a theory about the nurse’s role in neuro-rehabilitation of people who suffered a stroke in 1997 and revised it in 2010 by integrating newer research of the nursing role and function but also integrating experience-based knowledge from studies on the patient’s recovery and adjustment process (Kirkevold 1997, Kirkevold 2010). Her theory identified four therapeutic functions: ‘the conservative, the interpretative, the consoling and the integrative role and function in addition to a coordinating and leading function. Nurses facilitate bodily rehabilitation through conserving bodily functions, supporting the patients in continuing multiple therapies and helping patients interpret and integrate new learning skills into their everyday activities’ (Kirkevold 2010). Since this attempt, others have been made to further elaborate or expand on these descriptions and theories. In 2002, Long et al. undertook a two-year qualitative investigation in different rehabilitation settings and identified six interlinked roles for the nurse: assessment, coordination and communication, technical and physical care, therapy integration and therapy carry-on, emotional support and family involvement (Long, Kneafsey et al. 2002).  Kvigne et al. (2005) aimed to explore the nature of nursing care regarding the rehabilitation of female stroke survivors and found that nurses were focusing primarily on the functional and practical aspects of the women’s situations. In a systematic review and meta ethnography, Clarke (2013) aimed to create an explanatory framework for nursing practice in stroke rehabilitation. He identified that a nurse’s involvement in post-stroke rehabilitation was limited, further stating that the contribution was impacted by contextual factors, and the integration of rehabilitation skills was perceived to be contingent on adequate nurse staffing levels and management of demands on nurses’ time. Physical care and monitoring were found to be prioritized. The nursing role and function were found to be unclear, and the interdisciplinary collaborators and patients needed help to understand the role of the nurse in rehabilitation (Clarke, 2013).

In a literature review, Aadal et al. (2013) found that the four therapeutic roles and functions described by Kirkevold still reflected central aspects of current nursing stroke rehabilitation practice; however, they underscored the changes in practice related to newer patient roles and increasing interdisciplinary teamwork. Thus, because nurses are with the patients 24/7, they are well placed to perform rehabilitation activities and facilitate skills practice, assuming that the nurses have developed the appropriate knowledge of rehabilitation techniques and the techniques are regarded as legitimate nursing activities (Clarke, 2013).

Previous studies have highlighted the need for research on the development of interventions in the area of neuro-nursing (Clarke, 2014; Kirkevold, 2010). However, interventions aimed at optimizing the nursing staff’s contribution to inpatient rehabilitation are almost non-existent, and those that do exist document only modest effects (Clarke, 2013). This may be because the interventions are not sufficiently based on theory (Clarke, 2013).

Rehabilitation and nursing interventions are likely to be complex interventions as several components interact with those who deliver and those who receive the intervention. Developing an intervention that is aimed at strengthening the contribution of nursing staff in stroke rehabilitation has much to do with behavioural change (Craig et al., 2008). However, little attention has been paid to healthcare professionals’ behavioural changes in the development and implementation of complex interventions (Corry, Clarke, While, & Lalor, 2013). Interventions aimed at changing health professionals’ behaviour have generally proven problematic in showing their effect, possibly because of a lack of theoretical foundation and consideration in the development phase (Michie, Atkins, & West, 2014). Furthermore, interventions have been criticized for lacking a theoretical rationale and detailed reporting, thus complicating both the development and the possibility of replicating or improving interventions, and contributing to “research waste” (Michie, 2005).

The UK’s Medical Research Council (MRC) guidelines are useful for the development of complex interventions in health sciences (Craig et al., 2008). The MRC guidelines are in nursing the most commonly used in developing interventions (Corry et al., 2013) and are recommended for use in nursing research (Forbes, 2009). The MRC guidelines recommend that intervention development be theory- and evidence-based, but descriptions of how to do this are sparse. Michie et al. (2014) addressed this gap and developed an approach that integrates behavioural change theory into complex intervention design (Michie et al., 2014). The Behaviour Change Wheel (BCW) provides a useful framework for this. BCW is a theoretical model used to analyse behaviour. The BCW is based on the assumption that interactions between one’s capability (C), opportunity (O) and motivation (M) can explain why a particular behaviour (B) is or is not performed (COM-B) (Michie et al., 2014). The components are further subdivided. Capability may be physical (physical skills, strength or stamina) or psychological (knowledge or psychological skills, strength or stamina to engage in the necessary mental processes). Opportunity may be physical (opportunity afforded by the environment, time, resources, locations, cues, etc.) or social (afforded by interpersonal influences, social cues and cultural norms that influence the way we think). Motivation may be reflective (involving plans, self-conscious intentions or evaluations) or automatic (emotional reactions, desires, impulses, etc.) (Michie et al., 2014). The COM-B analysis guides the choice of intervention functions most likely to bring about behavioural change. The intervention functions have been linked to a taxonomy of 93 replicable behavioural change techniques (BCTs) (Michie et al. 2013). According to Michie, this structured approach lends transparency to the process of intervention development and facilitates subsequent implementation and evaluation (Michie, van Stralen, & West, 2011).

The BCW has been used in different settings within healthcare research. Within stroke care, we identified one study (Connell, McMahon, Redfern, Watkins, & Eng, 2015) using the BCW to develop an intervention aimed at increasing upper-limb exercise in stroke rehabilitation. To our knowledge, there are no published examples of mapping the BCW to MRC in developing an intervention aimed at strengthening the nursing staff’s contribution to inpatient stroke rehabilitation. As the application of the BCW may vary according to setting and target behaviour, we need examples of the generalisability of this approach. Furthermore, published examples of its use will contribute to the ongoing development and refinement of the method. In the present paper, we describe the development of an intervention to optimize the rehabilitation of patients after stroke by strengthening the role and functions of nursing staff in inpatient rehabilitation where we employed the BCW steps to enable more transparent development of the design and evaluation of complex interventions within the MRC framework.

## Methods

The MRC framework (Craig et al., 2008; Craig & Petticrew, 2013) for developing, evaluating and implementing complex interventions forms the basis of our study. It describes the development and evaluation of complex interventions as evolving in four phases—development, feasibility/piloting, evaluation and implementation—and should be understood as an iterative process (Craig et al., 2008). In this study, we focused on the development and feasibility testing of an intervention (the feasibility testing will be reported elsewhere). In order to support systematic, theory-based development of a behavioural change complex intervention, the stepwise guide BCW was mapped to MRC stages and applied (Michie et al., 2014). See Table I for a detailed description. To develop an intervention using the BCW is not only a guide, it is also a theoretical perspective using behavioural theory as a way to achieve the desired changes and developments in nurses’ role and function by addressing the nursing staffs’ Capability, Opportunity and Motivation

**Table I.** Description of the steps developing a complex intervention.

MRC development	BCW stages	BCW steps
1. Identify the evidence base: First, we reviewed the existing evidence on the nursing contribution to neurological rehabilitation and on rehabilitation for inpatients after stroke. We complemented this with new evidence to clearly define our problem of interest and then select and specify the behavioural targets for the intervention.	1. Understand the behaviour	1. Define the problem in behavioural terms: For this first step, we began searching for relevant literature. As a first priority, we chose systematic reviews that could help explain the complexity of and problems with the nursing role and functions in the rehabilitation of inpatient stroke survivors. From these, we found relevant information and identified gaps that were important to analyse further. On the basis of these findings, we conducted participant observation and interviews with the nursing staff and patients (reported elsewhere).
		2. Select the target behaviour: From the literature review, the interviews and study observations, we identified behaviours of nurses and nurse assistants related to their role and function in the rehabilitation of stroke survivors. Guided by the BCW, we selected key behaviours to target in the intervention. These criteria addressed the likelihood that behavioural change would be implemented, the likely impact of changing the behaviour, the spill-over effect on others’ behaviours and the ease with which each behaviour could be measured (Michie, 2014).
		3. Specify the target behaviour In this step, we specified the target behaviours as well as what and who needs to change, where and when, how often and with whom.
2. Identify/develop theory: In this phase, we used COM-B to develop a theoretical understanding of the target behaviours. This understanding guided the choice of intervention functions.	2. Identify intervention options	4. Identify what needs to change: An analysis of the target behaviours was performed using the COM-B model to establish what needed to happen for the two target behaviours to change. To understand the behaviours in the context in which they occurred, we looked at the nursing staff’s physical capability (e.g. having the physical skills to work with the patients’ goals), psychological capability (e.g. understanding the concept of rehabilitation), physical opportunity (e.g. having the possibility of documenting goal setting, progress, etc.), social opportunity (e.g. making it the culture for nursing staff to work actively and take co-responsibility for patients’ goals, reflective motivation (e.g. holding the belief that it is possible to integrate rehabilitation principles into daily care), and automatic motivation (e.g. having established routines and habits for working systematically with a rehabilitative approach). Furthermore, the theoretical domain framework (TDF) which consists of 14 domains was applied to an additional behavioural analysis.
		5. Identify appropriate intervention functions: In this step, we determined which intervention functions (education, persuasion, modelling, enablement, coercion, incentivisation, training, restriction, environmental restructuring) would be most likely to affect behavioural change in the intervention by mapping the individual components of the COM-B behavioural analysis onto the BCW linkage matrices. After this, we used the criteria of affordability, practicability, effectiveness and cost effectiveness, acceptability, side effects/safety and equity (APEASE) to assess potentially relevant intervention functions.
		6. Identifying policy categories: Was not relevant for this study
3. Model process and outcomes In modelling the intervention, we sought to identify and detail the intervention content by operationalising the former steps.	3. Identify content and implementation options	7. Identifying behavioural change techniques (BCTs) The identified intervention functions (step 5) constituted the foundation for linking the BCTs with the aim of finding the best way of serving the intervention functions and the mode of delivery appropriate to implementing the intervention. We used the links drawn between the BCW and the taxonomy of the 93 BCT, focusing especially on the list of those most frequently used to select BCTs. Then we assessed these BCTs according to the APEASE criteria together with the working group.
		8. Determine the mode of delivery To find the mode of delivery, we had to consider the who, what, how and where. We had to consider how this intervention could be delivered to the nursing staff in the context of a stroke unit and in what form. The content of the intervention, translating the BCTs into intervention content, was based on previous research and theory and was developed by the first author in collaboration with the last author and in consultation with the working group. The mode of delivery—the how and where—was discussed in the working group.

To inform the theoretical and evidence-based foundations of the intervention, we collected data using four different approaches:

1) Review of the literature. The literature was reviewed for 1) research related to the role and functions of nurses in inpatient stroke rehabilitation; 2) studies describing patients’ experiences with the rehabilitation process in the sub-acute phase after suffering a stroke; 3) existing intervention studies focusing on the contribution of nursing staff; and 4) research related to identifying relevant outcome measurements (will not be addressed in this paper). To structure the literature search PICO and PICo models were used (see Table II for a description of review strategy).We evaluated the methodological quality of the systematic reviews using Assessment of Multiple Systematic reviews (AMSTAR) (Shea et al., 2009). Based on the review over the literature we could conclude that there were gaps in the literature describing the nursing contribution and the patient’s experience of inpatient rehabilitation that we had to investigate further. Furthermore, we needed more knowledge about facilitators and barriers for the nursing contribution in inpatient stroke rehabilitation.

**Table II.** Search and review strategy based on the PICO and PICo models (Joanna Briggs Institute., 2011).

To identify relevant reviews, a structured literature search and selection was performed based on the PICO and PICo models; ● Population: Nurse and nurse assistants Phenomenon of interest: Role and functions in inpatient stroke rehabilitation Context: Anglophone and European countries ● Population: Patients with stroke Phenomenon of interest: Inpatient rehabilitation Context: Anglophone and European countries ● Population: Nurses and nurse assistants Phenomenon of interest: Interventions in inpatient stroke rehabilitation Context: Anglophone and European countries ● Population: Nurses and nurse assistants in stroke rehabilitation Intervention: Education or training Comparison: No education or training Outcome: No limits The literature searches were conducted in Medline, Cochrane, Scopus and Cinahl during the autumn of 2014 with supplementary searches during the development process. Inclusion criteria were: Any type of qualitative, quantitative or mixed methods study that reported patients’ and nursing staff’s experiences of inpatient stroke rehabilitation as well as studies reporting intervention studies within the area of inpatient stroke rehabilitation aimed at strengthening the role and functions of nursing staff. Exclusion criteria were: exclusive focus on outpatient rehabilitation, exclusive focus on patients’ experiences on “long term”, exclusive focus on informal caregivers’ perspective, interventions aiming at strengthening the nursing contribution through instrumental, pharmacological or “small exclusive focus” initiatives. The methodological quality of the reviews was assessed using the objective measurement tool, AMSTAR (Shea et al., 2009; Joanna Briggs Institute., 2011). Data were extracted to answer the following questions: How do patients experience the first stretch of time after suffering a stroke (while still admitted at the hospital)? What are the needs of patients who have suffered stroke during inpatient stroke rehabilitation? How do the patients experience care, support and collaboration with the nursing staff? What are the role and functions of the nursing staff in inpatient stroke rehabilitation? What are described as the major challenges for the nursing staff’s contribution? How have previous interventions sought to address the issue of nursing contribution? What have been described as effective mechanisms in previous interventions?

Field observation: The observations took place over one month during different shifts and days including weekends. Different situations were selected in which patients and nurses or nurse assistants interacted. The overall aim of the field observations was to explore nurses’ and nurse assistants’ beliefs, attitudes and actions related to their functions in an inpatient stroke rehabilitation unit and to gain knowledge of the structure and culture in the rehabilitation unit (Loft et al., 2017). Every session was audio-recorded and field notes were taken on the spot. The transcriptions were analysed using content analysis (Elo & Kyngäs, 2008; Graneheim & Lundman, 2004).

Semi-structured interviews: We conducted semi-structured interviews (Kvale & Brinkmann, 2009) with 12 members of the nursing staff employed at an inpatient stroke rehabilitation unit and 10 patients admitted to inpatient rehabilitation (Loft, Poulsen, et al., 2017; Loft, Woythal Martinsen, et al., 2017) . The interviews were transcribed and analysed using content analysis (Elo & Kyngäs, 2008; Graneheim & Lundman, 2004). The overall aim of the interviews with the nursing staff was to describe nurses’ and nurse assistants’ beliefs, attitudes and actions related to their functions in an inpatient stroke rehabilitation unit. The overall aim of the patient interviews was to describe patients’ experiences of inpatient stroke rehabilitation and their perceptions of nurses’ and nurse assistants’ roles and functions during hospitalisation.

The results from our own research were incorporated in the development of the intervention as both supporting the existing research but also elaborated and contributed with new and necessary knowledge.

2) Identifying relevant theory: We identified relevant theory based on the evidence found in the first steps to help support the effectiveness of mechanisms and actions in the intervention. The theory was selected in interplay between what was supported by evidence and pragmatic choices. For instance, the empirical theory of Marit Kirkevold relating to the nursing role and function in inpatient stroke rehabilitation was chosen to guide the understanding of the nursing contribution and as a foundation for the development of the intervention. This was chosen as the work of Kirkevold is considered a classical theory within the stroke area. It is often cited and a recent review concludes that it still accommodates central aspects of current nursing practices.

## Setting

Interviews and field observations which provided the empirical foundation for the intervention were undertaken at a stroke rehabilitation unit located at a university hospital in Denmark. The nursing staff consisted of registered nurses (*n *= 19) and nurse assistants (*n *= 18) with a mean seniority of 8.8 years (range 2–34 years) and a mean age of 44.6 (range 26–66 years). In the stroke unit, patients entered the acute clinic and if the patients were in need of in-hospital rehabilitation, they were transferred to the rehabilitation unit. The patients admitted for rehabilitation differed in complexity and severity of their conditions, and the length of their stay was dependent on this.

The first stages in developing the intervention (steps 1–6 in the BCW, Table I) were undertaken with the first and last authors of this paper taking the lead. From step 7, a working group was established that engaged three members from the nursing staff, two ward nurses, two professional advisers and the main researcher. The working group met approximately two times per month over 6 months. This working group was composed to ensure the development of a clinically relevant intervention that was tailored to the context. The two professional advisers we collaborated with, a Master of Science (MSc) in Economics and a Master in Organisational Psychology, contributed with knowledge of and experience in patient involvement, process improvement, change management as well as insider perspectives as former patient and as relative, respectively. Before the final intervention was feasibility tested, it was presented to a large group of interdisciplinary collaborators (physio-, occupational and speech therapists) who provided their input on the intervention.

## Ethical considerations

The entire study was registered with the Danish Data Protection Agency, file number H-2-2014-038. The ethics principles of the Declaration of Helsinki were followed.

## Results

### MRC stage 1: Identifying the evidence base

#### BCW step 1: Define the problem in behavioural terms

For this initial stage, we conducted a needs assessment that involved several steps.

##### The role and functions of nurses in inpatient stroke rehabilitation

First, we searched for literature related to the nurses’ role and functions in inpatient stroke rehabilitation. The criteria for selection are described in Table II. We identified 20 relevant articles, among them three (systematic) reviews and meta-ethnographies that covered the identified articles. From our search, we found that there were already good quality reviews on which we could draw covering the spectra of the original research. Hence, the three reviews were all included and served as the foundation for our need assesment. If we were uncertain about a need or behaviour identified in the review, we broadened our understanding by investigating the original article. In a systematic review and meta-ethnography, Clarke (Clarke, 2013) generated an explanatory framework for nursing practice in stroke rehabilitation, suggesting that stroke-specific education and training needs to be enhanced if nurses are to perceive that they have a role in rehabilitation (Clarke, 2013).

As they are with the patient 24/7, nurses are well placed to carry out rehabilitation activities and facilitate skills practice; this, however, assumes that nurses have developed knowledge of rehabilitation techniques and that these techniques are regarded as legitimate nursing activities (Clarke, 2013). However, the role and functions of nurses in stroke rehabilitation are poorly understood and have been difficult to describe (Aadal et al., 2013; Clarke, 2013; Kirkevold, 2010). Nurses are described as focusing on recovery and regaining lost functions (Kirkevold, 2010). Developing inter-professional teams in which nurses share full partnership requires focus on organization, leadership, management, staffing levels and environment (Clarke, 2013). The interdisciplinary collaboration between nursing staff and therapists has been described as complicated and characterized by a hierarchical relationship in which the therapist perceives his or her role as that of an expert. This hierarchical relationship contributes to nurses’ belief that rehabilitation is something that is distinct from care (Clarke, 2013).

Despite their poorly understood role, nurses believe that they have an important role in rehabilitation. The work is described as physically hard, and nurses are ambivalent about how and whether it is possible to integrate rehabilitation techniques in their care (Clarke, 2013). Skills related to basic care such as assessment, monitoring and wound care are prioritized. However, although these skills are important and necessary, they are not sufficient in themselves in stroke care where the principle of rehabilitation should underpin all activities. Part of the reason why nurses prioritize basic nursing care without integrating rehabilitation principles is lack of time to assess risk, prevent harm and maintain safety (Clarke, 2013). Furthermore, rehabilitation principles can conflict with nurses’ views on caring for patients. Nurses facilitate bodily rehabilitation through conserving bodily functions, supporting the patients in continuing multiple therapies and helping patients interpret and integrate new learning skills into their everyday activities (Kirkevold, 2010).

In a literature review, Aadal et al. (2013) found that the four therapeutic roles and functions described by Kirkevold in 1997 still reflect central aspects of current nursing practice, yet they underscored the changes in practice related to newer patient roles and increasing interdisciplinary teamwork. These reviews gave us an overall impression of what was at stake in nurses’ roles and functions in inpatient stroke rehabilitation, and they provided the foundation for the needs assessment.

##### Patients’ experiences with the rehabilitation process in the sub-acute phase after suffering a stroke

The second step involved a review of the literature to identify previous research describing the needs and experiences of patients admitted to rehabilitation after stroke, as these needs and experiences were important for us to know and integrate into the intervention for the process of literature search and process of selection, see Table II. We identified seven good quality reviews, meta-ethnographical and metasynthesis studies addressing patients’ needs from different angles following stroke. From those, we focused on three (Gallacher et al., 2013; Hole, Stubbs, Roskell, & Soundy, 2014; Satink et al., 2013) as they focused mainly on the first stretch of time after suffering a stroke while still being in inpatient rehabilitation, or where we were able to identify and differentiate findings in the articles that related to our area of interest.

Patients’ experiences of the aftermath of a stroke can be overwhelming and may be associated with physical, social and physiological consequences (Hole et al., 2014). Hole argues that healthcare professionals should consider their interaction and provide care that supports the patients’ psychosocial needs to help their change in identity (Hole et al., 2014). In line with this, Satink et al. (2013) found that stroke patients experience healthcare professionals as having views on how they should convalesce physically, while patients demanded more attention to their psychosocial needs and more agreement between the healthcare professionals on interventions and goals and the patients’ needs. Patients suffering a stroke may have to reconstruct a new self, and work hard to understand and acknowledge the ramifications of the stroke. Patients describe their human needs as not being met during rehabilitation and they feel that they are not acknowledged as individuals (Satink et al., 2013). Healthcare professionals are described as crucial for assisting in the process of rebuilding new lives, for instance by supporting and motivating patients (Hole et al., 2014). The patients demand autonomy, and some wish for an equal relationship and more shared decision-making with staff (Gallacher et al., 2013; Hole et al., 2014). However, some patients prefer a more paternalistic relationship where they rely on staff (Gallacher et al., 2013).

Patients experience hope and hopelessness as core concepts during their rehabilitation. Having hopes for the future sometimes collides with patients’ expectations of the amount of training they receive, and they find the staff’s role in their rehabilitation to be insufficient (Hole et al., 2014).

During inpatient rehabilitation, patients try to fit into routines. They can experience unfamiliarity with various gadgets; long waiting times for personal care; inadequate support during mealtimes; and lack of stimulating activities, privacy and dignity while on the ward (Gallacher et al., 2013). Goal-setting is a core feature of rehabilitation, and patients underline the importance of working with goals that are meaningful, which for patients often means reaching their former social status or role. Patients state that they experience a lack of support from staff in goal-setting. Positive experiences are shaped by key psychosocial concepts such as hope and social support, and rely on a sense of self-efficacy, which is influenced by both clinical staff and external support (Hole et al., 2014). Positive experiences appear to be associated with patients managing their conditions.

##### Existing intervention studies focusing on the nursing staffs’ contribution

As the third step, we searched the literature for nursing interventions in stroke care, focusing on educational interventions for nursing staff working in stroke rehabilitation that aim at enhancing nurses’ rehabilitative approaches, skills and professional identities. For the process of literature search and process of selection, see Table II. Previous studies have mainly focused on nursing interventions related to specific areas such as preventing further complications, swallowing, bladder problems, etc., or focusing on task-orientated training. As we aimed at enhancing the overall role and function of nursing staff working in inpatient rehabilitation, we therefore limited the search related to this issue. We identified four relevant studies, two of which were from before 2000. Due to the complexity of the rehabilitation and educational interventions which are not easy to randomize, we not only searched for randomized controlled (RCT) studies but also chose to search for quasi-experimental and intervention studies that were qualitatively evaluated. We identified one RCT from 2005 by Burton and Gibbon that aimed at expanding stroke nurses’ role. In this study, a training programme (the details were not specified) was provided for stroke nurses that aimed at providing continuity in care to stroke survivors after discharge to improve recovery from stroke. This is the only study identified which measured the effect on patient outcomes. Burton and Gibbon concluded that the intervention had substantial benefits for the patients. In 1998, Foster et al. reported on the effect of a physiotherapist-led training programme on the attitudes of nurses caring for patients after stroke. The intervention consisted of 9 h of training including lectures and interactive practical sessions, and the effects were measured using an attitude questionnaire and qualitative interviews. The authors concluded that the results indicated changes in the nurses’ attitudes to treating patients after stroke. In 1998, Jones et al. reported a quasi-experimental study which involved 2 h of classroom course aimed at improving nurses’ knowledge of and practice in the area of positioning. They concluded that the intervention had some effect. Booth et al. conducted a quasi-experimental study in 2005 that aimed at measuring the effect of a 7-h formal educational programme (lectures, simulated patient demonstration, video and experiential learning) that focused on therapeutic handling. They measured the effect using non-participant observation and concluded that a change in therapeutic style had occurred.

All of the above articles concluded that educational intervention had some effect on the nursing staffs’ approaches and attitudes toward rehabilitation, but as the interventions differed in content, duration and structure, it is impossible to conduct a meta-regression analysis to identify effective mechanisms, as recommended by the MRC framework. Hence, in the present study we do not base the intervention development on these very limited previous intervention studies, as the reporting quality and level of evidence is neither clear nor strong.

Based on the above review of the literature, we concluded that there was a need to develop an intervention that focused on strengthening the nurses’ role if stroke patients’ rehabilitation is to be optimized in a way that not only focuses on physical training, but also leaves room to address the patient as a whole while continuing rehabilitation 24/7. . Our aim was to improve the rehabilitative approach performed 24/7 by nursing staff in a stroke unit.

### BCW step 2: Select the target behaviour

We identified 69 candidate target behaviours that could promote the desired outcomes based on the literature review and the empirical study (see Table III). Based on established criteria (see Table I) rated with from one to four points in the guide, we immediately excluded seven of these behaviours, which were rated four points or ‘unacceptable to target’. An example of what was rated as unacceptable to target in the intervention was the ‘lack of bathrooms’ based on a field observation that identified this as a barrier for nursing staff and patients in daily care and rehabilitation practice. Furthermore, 20 of the target behaviours were rated with three points as “unpromising but worth considering”, and the final 42 were rated with one or two points as “promising” or “very promising”. From the final 42, we selected two target behaviours. The two target behaviours were based on a decision guided by knowledge gain from the literature review and own empirical study, but also considering what would be most pragmatic. The two selected target behaviours were: 1) working systematically with a rehabilitative approach, and 2) getting nursing staff to work deliberately and systematically with patients’ goals.

**Table III.** Sixty-nine candidate target behaviours.

● Barriers identified based on the literature review, field observations and interviews with nursing staff and patients
● Lack of rehabilitation skills ● Lack of training in rehabilitation skills ● Lack of understanding that nursing staff levels are not related to the possibility of integrating rehabilitation techniques ● Lack of respect for and awareness of nurses’ contributions to interdisciplinary team members (ITM) ● Lack of time to employ rehabilitation principles in daily care and practice ● Workload pressure that hinders possibilities to employ rehabilitation principles in daily care and practice ● Lack of understanding that direct care and monitoring provide opportunities to integrate rehabilitation principles ● Unclear nursing roles and functions in neurorehabilitation ● Disagreement on the ITMs see nurses’ coordinating role ● Difficulty securing RNs and NAs’ participation in education and training ● Differences in understandings of what care coordination means for caregivers and other ITMs ● Prioritization of physical care activity over rehabilitation principles ● A nursing culture of doing *for* instead of *with* patients ● Difficulty achieving interdisciplinary collaboration among the whole team in rehabilitation ● Difficulty performing joint work while shadowing more experienced ITMs, which is the best way to learn rehabilitative principles ● Inexperienced RNs and NAs’ perceptions of physical care as something different from rehabilitation ● Lack of confidence of all inexperienced ITMs in RNs and NAs’ ability to encourage and facilitate patients’ independence ● Lack of mandatory development of specific competencies in rehabilitation ● Changing nursing roles and functions during inpatient rehabilitation due to increased focus on individuality and patient-centred care ● Lacks of ownership of goals by RNs and NAs ● Failure of nursing staff who know patient goals to discuss them with patients ● Lack of communication between staff and patients about goals ● Nursing staff lack knowledge about long- and short-term goals ● Loss of patient motivation when staff members do not support rehabilitation goals ● Patients’ lack of knowledge about their own goals ● No sense of ownership and self-motivation in relation to rehabilitation goals among patients ● Patients feel unmotivated when they cannot see a purpose for goals ● Nursing staff’s lack of understanding of their own role in rehabilitation ● Patients have difficulties seeing nursing staff’s role in rehabilitation ● Nurses’ communication with patients lacks understanding of the task. ● No use of the conversation book (SCA) ● Lack of clarity among nurses about their roles and functions (belief that they are primarily administration and coordination, not rehabilitation) ● The career staff´s communication and personality affect how patients are rehabilitated ● Vague role of nurses in ward rounds ● Lack of encouragement from staff for patients to get out of bed and be active ● Effective self-training (both physical and cognitive) by patients but a demonstrated lack of encouragement and interest from both RNs and NAs ● Lack of focus and prioritization by RNs and NAs of sitting down with patients and asking how they feel ● Absent or rarely present consoling and interpretive role in rehabilitation ● Lack of continuity in the rehabilitation process ● Lack of prioritization of the contact person system ● Lack of systematic, consistent approaches to rehabilitation during the day ● Lack of constructive, professional communication ● Lack of knowledge of how to communicate with patients with brain injuries ● Lack of awareness of what positive communication means for patients ● Lack of knowledge about brain injuries ● Lack of knowledge about the concept of rehabilitation ● Little systematic or deliberate use of the integrative function ● Challenging physical environment ● Lack of bathrooms ● Lack of knowledge of how patients with brain injuries are involved in their rehabilitation ● Doing *for* instead of *with* the patients by RNs and NAs ● Lack of knowledge of own worth and opportunities to influence patients’ rehabilitation ● Lack of language to tell other ITMs what they (nursing staff) can do or are doing ● Difficulties in collaboration between RNs and therapists ● Difficulties in collaboration between NAs and therapists ● Difficulties in collaboration between RNs and NAs ● Numerous interruptions during care sessions ● Limited understanding of rehabilitation as a similar concept as physical rehabilitation ● Absence of a culture for searching knowledge and evidence ● Lack of documentation of patients’ rehabilitation process and progress towards goals ● Low prioritization of rehabilitation ● Lack of professional leadership ● Difficulty working with concrete goals ● Lack of understanding that integrating rehabilitation principles into daily care is not time consuming ● Lack of understanding that working with patients goals in daily care is not time consuming ● Negative understanding of own role (nursing staff) ● No perception by patients that RNs and NAs are part of their training and rehabilitation ● No implementation of working with goals in patients’ daily care

### BCW step 3: Specify the target behaviour

The two target behaviours were each specified according to who needs to do what, when, where, how often and with whom. With regard to both target behaviours, we needed all the nursing staff to perform the behaviour in the stroke unit, 24 h a d all week long, either alone, with the interdisciplinary collaborators or with the patient. Some of the behaviours listed below could be said to be related to organizational factors influencing practice and how nurses conceptualize these. However, behaviours occur within a system of other behaviours, thereby the way the nursing staff conceptualize this is related to their behaviour. The way to specified behaviours, as displayed below, will be important in determining how far the problem is solved.

For the first target behaviour, “working systematically with a rehabilitative approach”, nursing staff needed to change the following behaviours to achieve the desired change:

- Understanding that nursing staff levels are not related to the possibility of integrating rehabilitation techniques
- Understanding that direct care and monitoring provide opportunities to integrate rehabilitation principles
- Understanding that integrating rehabilitation principles into daily care is not (necessarily) time-consuming
- Being aware of one’s own role and worth, and of the opportunity to influence the patient’s rehabilitation
- Being more assured of one’s own role and function
- Doing with instead of for the patient
- Changing priorities so that physical care activity does not take priority over rehabilitation

For the second target behaviour, “getting nursing staff to work deliberately and systematically with the patient’s goals”, nursing staff needed to change the following behaviours to achieve the desired change:

- Taking ownership of goals together with interdisciplinary collaborators and the patient
- Documenting the process and progress
- Talking with and involving patients systematically in the goal (setting) work, every day and during every shift
- Communicating with colleagues and interdisciplinary collaborators about the process and progress
- Making sure always to get to know the patients’ goals—both long-term and short-term ones—before starting the care session
- Prioritising the contact person system
- Prioritising continuity in the care trajectory

## MCR phase 2: Identifying/developing theory

### BCW step 4: Identify what needs to change to achieve the desired behaviours

The COM-B model was used to identify nurses’ and nurse assistants’ capabilities (C), opportunities (O) and motivations (M). Analysing the two target behaviours using the COM-B model helped in understanding these two target behaviours within the context in which they occur. To broaden the understanding of the behaviour and to improve the implementation of the intervention the Theoretical Domains Framework (TDF) was applied. The TDF consists of 14 domains, which can be related to the COM-B components (Michie et al., 2014). Table IV shows an example of the results from this analysis and how the barriers are linked to the COM-B factors.

**Table IV.** Example of barriers linked to COM-B, TDF and intervention functions.

COM-B	BARRIER	TDF	INTERVENTION FUNCTIONS
Psychological capability	Lack of knowledge of how goalsetting influences motivation and outcomes for the patient	Knowledge	Education
	Lack of skills in collaboration	Cognitive and interpersonal skills	Training
	Lack of attention to the patient	Memory, attention and decision processes	Training, environmental restructuring enablement
	Lack of skills that make it possible to apply if-then rules	Behavioural regulation	Education Training Modelling Enablement

### BCW step 5: Identify intervention functions to achieve the desired behaviour

To maximize the nursing staff’s capabilities and to increase their motivation to change their behaviours related to working with a rehabilitative approach and working deliberately and systematically with patients’ goals, we concluded that the intervention should include the following seven intervention functions: education, persuasion, modelling, incentivisation, training, enablement and environmental restructuring (Table V). The criteria of affordability, practicability, effectiveness, and cost effectiveness, acceptability, side-effects/safety, and equity (APEASE) helped to assess the potentially relevant intervention functions.

**Table V.** Intervention content and mechanisms of action using the Behaviour Change Model, and TDF.

	Intervention content		Mechanisms of action	
Intervention component	BCTs	Functions	COM-B	TDF
**Group education:**				
Information on and discussion about rehabilitation and the nursing staff’s role and functions in inpatient stroke rehabilitation and their significance for patients’ rehabilitation	Information about health consequences, Information about social and environmental consequences	Education	Psy C, Ref M	Kn, Id
Information on and discussion about health consequences for the patient of working systematically with the patient’s goals, e.g. the evidence behind	Information about health consequences	Education	Psy C	Kn
Information on and discussion about patients’ experiences of suffering a stroke and subsequent rehabilitation	Information about health consequences, Information about social and environmental consequences	Education	Psy C	Kn
Establishing a method for the nursing staff to monitor and record their behaviours as part of a behavioural change strategy.	Feedback on behaviour	Education	Psy C, Ref M	Kn, B Con
Instruction on how and when to work with a rehabilitative approach	Instruction on how to perform the behaviour	Education	Psy C	Kn
Stories of patients who had experienced inpatient rehabilitation	Information about health consequences	Education, Persuasion	Ref M, Auto M	B Con, Em,
Evidence of inpatient rehabilitation, patient outcomes given, and patients’ experiences	Information about health consequences	Education, persuasion	Psy C, Ref M	Kn, B Con
Presentation of and discussion of feedback (presentation given as part of the kick-off by a *celebrity)*	Credible source	Education Training Incentivisation	Psy C, Ref M Aut M	Kn, Cog Id, Em
Discussion of the effects of good rehabilitation on others and own wards	Social comparison	Persuasion	Soc O, Ref M	SI, B Cap, Id
Discussion on time and staffing levels in relation to the possibility of working with a rehabilitative approach	Verbal persuasion about capability	Persuasion	Ref M	B Cap, Opt
**Material and exercises:**				
Non-participant observation of own practice	Demonstration of behaviour, instruction on how to perform behaviour, behavioural practice, habit formation, feedback on own behaviour	Modelling, persuasion, education, training	Phys C, Psy C Ref M	Sk, Kn, MAD, BR, B con
Video provided instruction on working with a rehabilitative approach	Instruction on how to perform behaviour	Education, persuasion, incentivisation, modelling	Psy C, Ref M, Auto M	Kn, B Con, Em, Reinf.
Poster visualising the participants’ individual goals, goals for the group/unit, subjects of discussion and agreements	Feedback on behaviour, prompts/cues, goal setting, problem solving, action planning	Education, Enablement	Psy C, Ref M,	Kn, Mem, Goals, Id
Log book	Self-monitoring of behaviour	Education Persuasion incentivisation	Psy C, Ref M	Kn,Int, Goal
Stickers for the log-book illustrating the main points from the course, explanations of the task, etc.	Prompts/cues	Education, Modelling	Psy C, Ref M, Auto M	Env
Labels on the overview notes	Self-monitoring, prompts/cues	Enablement, environmental restructuring	Psy C	MAD, BR, Env
**Feedback and how to continue implementing**				
Verbal feedback on individual performance given	Feedback on behaviour	Education, training, persuasion	Psy C, Ref M	Kn, B Con
Group discussions about the successes brought about by working on one’s goals and what it meant for the patient/oneself	Feedback on behaviour, feedback on outcome of the behaviour	Education, training, persuasion	Psy C, Ref M	Kn, B Con
Generating solutions for implementation (for the last workshop)	Problem solving, action planning, goal setting, restructuring the physical environment	Enablement	Psy C, Soc O, Ref M	BR, Id, B Cap

TDF domain abbreviations: Sk skills; Kn knowledge; MAD memory, attention and decision processes; BR behavioural regulation; SI social influences; Env environmental context and resources; Reinf reinforcement; B Cap beliefs about capabilities; B Con beliefs about consequences; Id social/professional role and identity; Opt optimism; Goal goals; Em emotions; Cog cognitive and interpersonal skills. COM-B abbreviations: Phys C physical capability; Psy C psychological capability; Soc O social opportunity; Phys O physical opportunity; Ref M reflective motivation; Auto M automatic motivation

### BCW step 6: Policy categories

Although we were not primarily concerned with changing policy in this study we for future interest carried out the analysis related to policy categories and found guidelines, regulation and legislation useful for achieving behavioural change.

## MRC stage 3: Modelling process and outcomes

### BCW step 7: Identify behavioural change techniques

At this stage, we were concerned with finding the key components for the intervention and identifying which BCTs best served the intervention functions (Michie et al., 2014). The taxonomy of 93 BCTs were listed and compared with the seven intervention functions we had chosen. To begin with, we looked especially at the most frequently used BCTs and then shortened the list using the APEASE criteria. We then looked more closely into the definitions of the relevant BCTs and brainstormed on how they could be operationalized in the intervention by considering previous research and the new empirical evidence. An example of this was the BCT feedback on the outcome of the behaviour, a BCT linked to the function “persuasion”. We knew from the literature and the empirical evidence that nursing staff face difficulties envisioning their own role and functions in rehabilitation; we also knew, for example, that patients described not feeling supported by the nursing staff. A suggestion for operationalising this BCT was to get the staff to make patient-centred observations. From previous research and theory, we know that observations from the patient’s perspective can provide insight into how the system works in ways that cannot be discovered by simply reflecting on one’s own experience and practice (Bisgaard & Ebdrup, 2012; Elwyn et al., 2012; Graban, 2009). Patients’ experience in healthcare is closely linked to their physical presence: where they are, how they feel, their relationships and what is happening around them. Through observations, nursing staff can reflect on the quality of the care from the patients’ perspective. In many cases, this is different from the quality judged by professional standards and guidelines. If there is a significant difference between nursing staff’s own understanding of the care provided and the way it operates in practice, an attentive observer can obtain an impression of the differences. Since successful rehabilitation depends largely on the patient’s motivation to train, it is especially important to look for how nurses affect patients’ hopes and their desire to participate in personal care and other routine activities during their hospital stays. Observers can notice their colleagues’ behaviour and use this to reflect on how they themselves are experienced in their own professional roles. Each relevant BCT was addressed in this way, which made it possible for us to draft an intervention strategy.

### BCW step 8: Identify mode of delivery

Finally, we formulated an intervention plan and planned how it should be delivered. The intervention was delivered as a seven-week educational programme for nursing staff and broadly consisted of the following components: group education and training (face to face), training in practice (individual and with reflection partner) and materials (log book) provided partly as a feedback tool, partly as a reflection tool and partly as educational material.

The intervention was delivered to all the nursing staff working in the rehabilitation unit including ward nurses. They were purposively divided into three groups. We wanted to split up normal alliances in the staff group; furthermore, we wanted to distinguish by age, degree of experience, competence and educational level. During the seven-week programme, each group had three group sessions of 3 h each. Between the sessions, the staff had tasks and training to work on in their daily practice. The intervention (education) was delivered by members of the working group with the main researcher in charge. The following criteria were set for the intervention delivery (at least two people and one of each):

1. Having a nurse with at least a master’s degree.
2. Having another professional with at least a master’s degree who had experience in facilitating processes of change in the healthcare system and competency development.

In Table VI, examples of the general content of the intervention inspired by the TiDerR framework (Hoffmann et al., 2014) are summarized.

**Table VI.** Rehabilitation 24/7 based on TIDieR.

Intervention components	Rationale	Mode of delivery	Delivered to	When/how often
Educational sessions ● Introduction ● Rehabilitation (concept, history, rationale, definitions, evidence, patients’ narratives) ● Feedback	To familiarise the staff with the concept, increase knowledge and motivate and generate enthusiasm to change own practice	Face-to-face (group)	Nursing staff	Three times for three hours as a kick off and then with two-week intervals
Training of different tasks related to rehabilitation and exercises (e.g. verbalising own role and function in rehabilitation)	To train staff in a secure context with the possibility of direct feedback and guidance	Face-to-face (group)	Nursing staff	Integrated as part of the educational sessions
Training in practice (e.g. training own goals for behavioural change)	To train staff in a rehabilitative approach in a real-life setting	Individual or face-to-face with reflection partner	Nursing staff	Ongoing in the seven week programme
Educational documents	To educate staff about rehabilitation and nursing staff roles and functions, and promote self-monitoring	Documents	Nursing staff	Ongoing in the seven-week programme
Observation of own practice	To motivate, increase knowledge and incentivise participants to feel positively about the desired behavioural change	Individual (face-to-face in reflection and subsequent analysis)	Nursing staff	Two-four hours in the second week of the educational programme
Audit and group feedback—daily prompts and cues at *the day overview note*, posters in the staff area and verbal feedback given by the leaders and stakeholders	To focus staff’s attention on targets and progress	Feedback delivered face-to-face (group)	Nursing staff	Ongoing in the seven-week programme

## Discussion

The aim of this article was to describe the systematic, structured development of an intervention to optimize the rehabilitation of stroke inpatients by strengthening the nursing staff’s role and functions. Findings from previous research literature that addressed the nursing staff’s role and functions in inpatient rehabilitation, the needs of stroke patients during inpatient rehabilitation and previous intervention studies within this area were key to the development of the intervention. These elements were supplemented empirically by field observations and interviews. The BCW guided the development through its eight steps.

We were guided by the MRC framework in developing a complex intervention. Richards and Borglin argue that nursing and nursing interventions are complex and that designing nursing intervention studies is difficult due to the complex organizational structure and multiple forms of behaviour within nursing practice (Richards & Borglin, 2011). The MRC framework, which has been found suitable for developing healthcare interventions, describes the development, testing, evaluation and implementation of interventions as four separate, iterative steps. Nursing interventions have previously been criticized for being underdeveloped and for having undocumented effects. Among nursing and other healthcare researchers, there is the tendency to argue that a systematic and transparent theory-and-evidence-based approach can hinder so-called research waste. Research waste is due to researchers asking the wrong questions, using unnecessary or poor-quality research methods, failing to publish research and reports, and deriving findings in a biased manner (Chalmers & Glasziou, 2009; Richards & Hallberg, 2015). The MRC framework also describes the risk of getting a promising intervention rejected as inefficacious due to insufficient effort having been made to develop and pilot it before a full-scale study is undertaken (Richards & Hallberg, 2015).

We began this study with the broad aim of developing an intervention aimed at optimizing the rehabilitation of patients hospitalized with stroke by strengthening the nursing staff’s role and functions, but we did not have a predefined idea of what the intervention would be. The MRC guideline of employing a theoretical approach, which was chosen *a priori*, provided direction, structure and transparency to this process in multiple ways. First, the MRC notes the need to identify the evidence base and to supplement this with new evidence if necessary. We identified literature reviews based on qualitative studies; however, we found no appropriate experimental studies useful for the present purpose. We were therefore unable to comply with the recommendations by Hallberg and Richard (Richards & Hallberg, 2015) to carry out regression analysis to identify components that promoted efficiency. This illustrates the challenges of developing complex interventions within the MRC framework in a field characterized by mostly qualitative and descriptive research and no or only few previous intervention studies related to the subject. We addressed this issue by developing the intervention, guided by COM-B.

Secondly, we used empirical data to directly influence the development of the intervention. The BCW steps allowed us to develop a list of options for behavioural change and to clarify what we were and were not trying to achieve.

In previous research using the BCW framework, researchers also used interviews, field observation and/or questionnaires to identify factors that need to change in order for the desired behaviour to occur (Mc Sharry, Murphy, & Byrne, 2016; Steinmo, Fuller, Stone, & Michie, 2015). In our study, we supplemented this approach, as recommended in the MRC, with knowledge from systematic reviews. This strengthens the likelihood of generalisation as the intervention is based on a broader perspective that includes different countries and settings.

Despite the highly systematic and structured approach of the BCW and the MRC frameworks, their use involves challenges. For example, the researcher needs to make a number of subjective and pragmatic decisions throughout the process. These decisions can seem at odds with a scientific approach. Hence, to improve the transparency and generalisability of our methods, we recorded in detail the multiple options available to us at each step of the BCW and MRC and clarified why options were or were not taken.

Furthermore, going through the many steps involved in developing the intervention was a lengthy process: it took two and a half years from the initial steps of reviewing the literature to making final refinements of the intervention. Such a long stretch of time is resource-demanding; it is a factor that needs to be considered both by those conducting and those funding evidence-based intervention development. Hallberg (Richards & Hallberg, 2015) argues that research should take place within a research programme (Richards & Hallberg, 2015). Doing research within a research programme facilitates deepening of the problem area and illuminating the problem area from several angles using different methodological approaches. In our study, this was not an option. However, it would have strengthened the study if we had had the possibility of moving back and forth between the development and feasibility/piloting phase.

We chose to develop this intervention within the context of a working group consisting of members who brought different perspectives to the table. This, we believe, strengthened the development of the intervention as the group could collectively contribute with perspectives beyond our own perspectives as researchers. It could be queried whether the working group should have been involved at an earlier stage and what this would have meant for the intervention. In future work, further patient and public involvement should be integral to improving the intervention. Furthermore, it could be questioned if the working group should have included allied health professionals, as inpatient stroke rehabilitation takes place in an interdisciplinary context. However, as the literature displays the nursing role and function as vaguely defined opposite their interdisciplinary colleagues and the collaboration is described as challenging (Loft, Poulsen, et al., 2017; Luker et al., 2016), we had considered it appropriate with the chosen working group to minimise the risk of barriers on this basis lest it should hinder the learning objectives. Also, for good interdisciplinary collaboration it is important to provide different but complementary knowledge, skills, and aptitudes, and mutual respect for the other team members (Neuman et al. 2010) (Johnson, 2015). The contribution in the interdisciplinary teamwork must be based on one’s own skills and awareness of professional expertise (Neuman et al., 2010) (Johnson, 2015) and if the nursing staff is unsure of their own role and function as displayed this must be the first step to strengthen. However, we do acknowledge the important issue on where in the process to integrate the allied health professionals, as well as strengthening inpatient stroke rehabilitation as a composite effort.

We employed a “bottom-up” approach to theory development in which the frameworks of the BCW and MRC guided our use of existing evidence and our own qualitative explorations. This led to an intervention that was logical and practical, yet theoretically based. The next step is then to test the educational programme for its feasibility and acceptability and furthermore investigate the nursing staff’s self-perceived outcome of the educational programme in relation to the two target behaviours. These studies should inform us about any possible changes important to integrate into the intervention as well as informing us about possible changes in relation to the implementation strategy before a future effect trial can be launched.

Using a systematic, step-by-step approach, we developed the intervention called Rehabilitation 24/7. We exemplify how to map the BCW to the MRC framework when developing a complex intervention. Notwithstanding the challenges:time-consuming, complexity and many pragmatic decisions in the developmental phase, the systematic, transparent results that we have presented in this paper will facilitate a thorough evaluation of the intervention’s effectiveness and, after a feasibility test, make it possible to establish which of the key components are and are not working.
